# Assessment of chiropractic care on strength, balance, and endurance in active-duty U.S. military personnel with low back pain: a protocol for a randomized controlled trial

**DOI:** 10.1186/s13063-018-3041-5

**Published:** 2018-12-05

**Authors:** Robert Vining, Amy Minkalis, Cynthia R. Long, Lance Corber, Crystal Franklin, M. Ram Gudavalli, Ting Xia, Christine M. Goertz

**Affiliations:** 1 Palmer Center for Chiropractic Research, Palmer College of Chiropractic, 741 Brady St, Davenport, IA 52803 USA; 2 College of Chiropractic Medicine, Keiser University, 2081 Vista Parkway, West Palm Beach, FL 33411 USA; 3 Mechanical Engineering, Northern Illinois University, 590 Garden Rd, DeKalb, IL 60115 USA; 4 The Spine Institute for Quality, 102 2nd Street, Davenport, IA 52801, USA

**Keywords:** chiropractic, spinal manipulative therapy, military, strength, balance, endurance, low back pain

## Abstract

**Background:**

Low back pain (LBP) is a common cause of disability among U.S. military personnel. Approximately 20% of all diagnoses resulting in disability discharges are linked to back-related conditions. Because LBP can negatively influence trunk muscle strength, balance, and endurance, the military readiness of active-duty military personnel with LBP is potentially compromised. Chiropractic care may facilitate the strengthening of trunk muscles, the alteration of sensory and motor signaling, and a reduction in pain sensitivity, which may contribute to improving strength, balance, and endurance for individuals with LBP. This trial will assess the effects of chiropractic care on strength, balance, and endurance for active-duty military personnel with LBP.

**Methods/design:**

This randomized controlled trial will allocate 110 active-duty military service members aged 18–40 with non-surgical acute, subacute, or chronic LBP with pain severity of ≥2/10 within the past 24 h. All study procedures are conducted at a single military treatment facility within the continental United States. Participants are recruited through recruitment materials approved by the institutional review board, such as posters and flyers, as well as through provider referrals. Group assignment occurs through computer-generated random allocation to either the study intervention (chiropractic care) or the control group (waiting list) for a 4-week period. Chiropractic care consists primarily of spinal manipulation at a frequency and duration determined by a chiropractic practitioner. Strength, balance, and endurance outcomes are obtained at baseline and after 4 weeks. The primary outcome is a change between baseline and 4 weeks of peak isometric strength, which is measured by pulling on a bimanual handle in a semi-squat position. Secondary outcomes include balance time during a single-leg standing test and trunk muscle endurance with the Biering-Sorensen test. Patient-reported outcomes include pain severity, disability measured with the Roland Morris Disability Questionnaire, symptom bothersomeness, PROMIS-29, Fear Avoidance Beliefs Questionnaire, expectations of care, physical activity, and global improvement.

**Discussion:**

This trial may help inform further research on biological mechanisms related to manual therapies employed by chiropractic practitioners.

**Trial registration:**

ClinicalTrials.gov, NCT02670148 Registered on 1 February 2016.

**Electronic supplementary material:**

The online version of this article (10.1186/s13063-018-3041-5) contains supplementary material, which is available to authorized users.

## Background

Low back pain (LBP) is a prevalent and burdensome health problem in military populations [1–4]. More prevalent in active-duty military personnel than other populations, LBP is also a common cause of disability among those in combat deployments [4]. Approximately 20% of diagnoses resulting in disability discharges from the U.S. military are for back-related musculoskeletal conditions [5]. For those who are not disabled, LBP negatively impacts mission readiness by compromising the fitness of the individuals who make up deployable units [6, 7].

Fitness comprises many factors, such as strength, balance, and endurance, and these can be affected by LBP [8–11]. Poor trunk muscle strength may be a risk factor for future LBP [9], which results in altered muscle activity or movement patterns to avoid or prevent pain [12, 13]. Trunk muscle endurance, which facilitates prolonged or repeated positions or movement [14], can become compromised in individuals with LBP [11]. In individuals with LBP, balance may also be compromised by disturbances within sensory or motor components of the neuromuscular system [15, 16].

Chiropractic practitioners are trained health-care professionals whose focus is on the diagnosis and management of musculoskeletal disorders, especially those involving the spine [17]. Spinal manipulation (SM), among other nonpharmacological therapies used by chiropractic practitioners, is recommended as a first-line treatment for LBP by the American College of Physicians [18, 19]. As with most manual therapies, the biological mechanisms underlying chiropractic care (CC) are complex and only partially understood. SM may facilitate the strengthening of trunk muscle through multiple mechanisms [20], including motor neuron facilitation or disinhibition [21, 22] and by increasing trunk muscle thickness [23, 24]. SM procedures alter sensory signaling, which may influence changes in motor output [20, 25], and reduce pain sensitivity [26–28]. Trunk muscle strength may be the most important factor for improving function in individuals with LBP because it is a basic muscular characteristic necessary for balance and endurance.

To understand better whether CC influences strength, balance, or endurance in active-duty individuals with LBP, the United States Office of Congressionally Directed Medical Research Programs funded this Defense Health Program Chiropractic Clinical Trial Award (W81XWH-11-2-0107). The objectives of this randomized controlled trial (RCT) are to assess the effects of CC on strength, balance, and endurance for active-duty military personnel with LBP.

### Specific Aims

The primary aim of this clinical trial is to compare the effects of 4 weeks of CC versus a waiting list control group on strength in active-duty service members. Secondary aims are focused on estimating the effects of 4 weeks of CC versus a waiting list control group on balance and endurance.

## Methods/design

### Overview

This protocol describes a single-site RCT conducted at the Naval Air Technical Training Center branch clinic at Naval Hospital Pensacola, Florida (NHP). In total, 110 active-duty military personnel stationed at NHP with chronic, subacute, or acute non-surgical LBP are being allocated. Eligible participants are allocated to one of two groups, CC or a waiting list. Primary outcome measures include assessments of strength, balance, and endurance. Patient-reported outcomes (PROs) are also collected to assess pain intensity, LBP symptomology, functional status, and fear avoidance behavior. PROs are collected at baseline and at 4 weeks post allocation. This manuscript follows the recommendations of the Standard Protocol Items: Recommendations for Interventional Trials (SPIRIT) (Additional file 1).

**Fig. 1:**
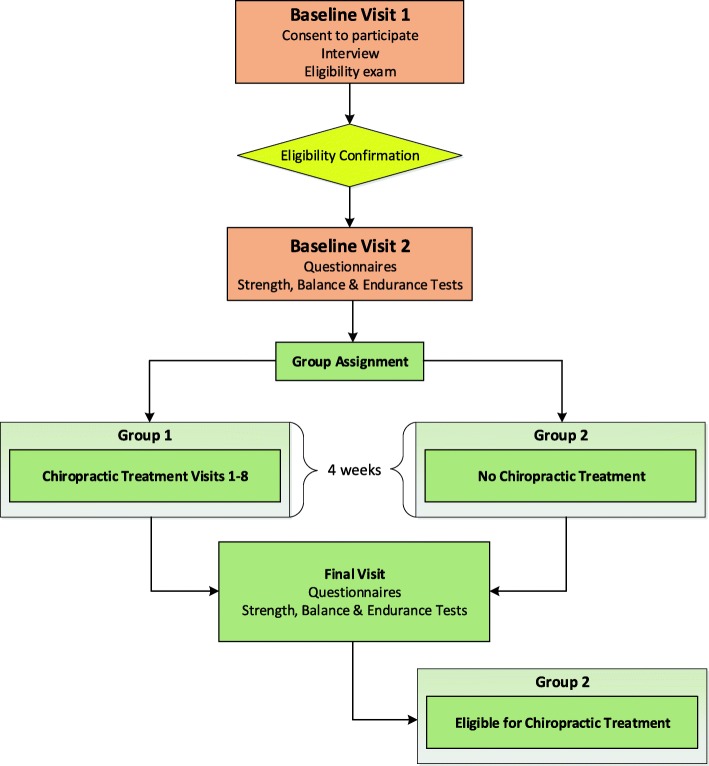
Study flowchart

Strength, balance, and endurance are assessed prior to allocation and again at 4 weeks. All functional and PRO measures are collected prior to any treatment that may occur on the same day. Figure 1 is a schematic flow of the trial protocol.

The trial utilizes the Submission Tracking and Reporting System (STaRS), a comprehensive web application developed and maintained by the Office of Data Management & Biostatistics within the Palmer Center for Chiropractic Research (PCCR). STaRS is a secure electronic data capture and clinical trial management system. Trial staff use STaRS for data entry, to confirm participant eligibility, to access real-time reports for tracking visits, and to report trial events.

### Trial organization

The investigative team for this RCT has members from three collaborating institutions: the RAND Corporation, PCCR, and the Samueli Institute. The RAND Corporation is responsible for grant administration and financial aspects of the award. RAND also functions as the communication link between the trial team and the program officer at the U.S. Department of Defense. The Samueli Institute provides advice regarding processes related to conducting research within military settings.

The PCCR investigators developed, implemented, and are managing the RCT at NHP. A site project manager (PM) at the Naval Air Technical Training Center branch health clinic is responsible for the everyday implementation of the project, including site-level documentation, recruitment, enrollment, adverse event recording, participant tracking, communicating with participants, and collecting data for the strength, balance, and endurance measures. One United States Navy medical physician serves as the site project investigator and another as the medical monitor.

A lead PM at the PCCR oversees trial operations, serves as liaison between trial investigators at RAND and the Samueli Institute, monitors progress, and ensures protocol adherence. The lead PM at PCCR and a central trial clinician (also at PCCR) review and monitor adverse events. Adverse events are graded by the central trial clinician. The PCCR clinical trial team meets regularly to review and monitor progress, discuss issues, and develop resolution plans.

The military health-care clinic where the trial is conducted has treatment space for one chiropractor. Other health services at the branch clinic include dentistry, primary care, sports medicine, physical therapy, radiology, and a pharmacy. As a branch clinic of NHP, care is available to approximately 25,000 active-duty military personnel, primarily members of the U.S. Navy and Marines. Chiropractic services have been available at the clinic since September 2003. The most common treatment is SM for neuro-musculoskeletal conditions, usually involving the spine. The clinic also includes a separate space to conduct strength, balance, and endurance measures. To date, two chiropractors have provided chiropractic services for the trial. Both have a Doctor of Chiropractic degree, an additional Master’s degree, and over 20 years of clinical experience. However, an additional graduate degree is not a requirement for employment at NHP. At NHP, as in other U.S. Naval treatment facilities, civilian chiropractors are employed through a subcontractor.

### Data and safety monitoring committee

A data and safety monitoring committee (DSMC) oversees this RCT. All members are independent of the collaborating institutions involved in conducting the trial. DSMC responsibilities include reviewing biannual reports to: (1) ensure participant safety and (2) advise the principal investigators (PIs) regarding scientific and ethical conduct. The DSMC evaluates adverse event data, protocol deviations, reasons for exclusion, and participant accrual. DSMC members make recommendations to the PIs and the Department of Defense regarding continuation, termination, or other modifications of the RCT.

### Regulatory approvals

The trial protocol received ethics approval from institutional review boards (IRBs) at the following three organizations: Palmer College of Chiropractic (2015G171), the RAND Corporation (2013–0159), NHP (NHPC.2015.0003, IRB of record: Naval Medical Center Portsmouth, VA). The trial protocol was also approved by the U.S. Army Medical Research and Material Command Human Research Protection Office and the Naval Medical Center Portsmouth Clinical Investigation Department. All investigators completed training on the protection of human subjects as required by the respective institutions.

### Recruitment procedures

We are recruiting 110 active-duty military personnel, ages 18–40, with self-reported non-surgical, acute, sub-acute, or chronic LBP with pain severity of ≥2/10 within the past 24 h. Full descriptions of the eligibility criteria are given in Table 1. Participants are recruited through IRB-approved recruitment materials such as posters and flyers as well as through provider referrals. Other methods of recruitment include use of the military base intranet to advertise the trial. In addition to IRB approval of recruitment activities, command permission was obtained prior to recruitment, from the NHP Commanding Officer, to post IRB-approved advertisements and recruit participants.

**Table 1:** Eligibility criteria

Criteria	Rationale
Inclusion criteria	
Age 18–40 years	Age range of most active-duty military personnel
Able to provide voluntary written informed consent	Able to comprehend trial details and make independent decisions without impairment
Active-duty status at Naval Air Station Pensacola	Chiropractic care and assessment instruments available only to active-duty personnel at Naval Air Station Pensacola
Self-reported acute, subacute, or chronic low back pain severity at initial screen and baseline visit 1 with a pain intensity of ≥2 within the past 24 h on a 0–10 numerical rating scale	Low back pain with enough severity likely to demonstrate reduced strength, balance, or endurance
Exclusion criteria	
Any chiropractic care within 30 days prior to informed consent	Prevent possibility of carryover effects from recent care
Low back pain confirmed or suspected to arise from a visceral source	Evaluation or care outside trial scope required
Spinal pathology or other condition contraindicating spinal manipulation	Evaluation or care outside trial scope required
Spinal fracture within the past 6 months	To ensure safety in strength, balance, and endurance tests
Spinal surgery within the past 6 months	To ensure safety in strength, balance, and endurance tests
Low back pain with presumptive or confirmed spinal nerve root compression	To ensure safety in strength, balance, and endurance tests
Imaging evidence of neuroforaminal or spinal canal stenosis or the clinical presentation of neurogenic claudication	To ensure safety in strength, balance, and endurance tests
Chronic pain syndrome diagnosis	Condition not under study and evaluation or care outside trial scope required
Diagnosis or suspicion of inflammatory spinal arthropathy	Condition not under study and evaluation or care outside trial scope required
Referral needed to evaluate or treat an urgent or emergent condition or to determine status of a condition pertaining to eligibility	Evaluation or care outside study scope
Unable to perform strength, balance, or endurance tests safely, or unable to assess health status	To ensure safety in strength, balance, and endurance tests
Pregnancy or planning to become pregnant within the next 6 weeks	To ensure safety in strength, balance, and endurance tests and to avoid confounding assessments due to changing physical status during pregnancy
Knowledge of impending transfer or absence during trial period	To ensure ability to comply with trial protocol
Seeking or referred to medical evaluation board or physical evaluation board regarding disability status	To ensure safety in strength, balance, and endurance tests and to ensure ability to comply with trial protocol
Unable or unwilling to comply with trial protocols	To ensure able to comply with trial protocol
Patellar height is <15 in. (38.1 cm) or >25.5 in. (64.8 cm)	To ensure strength assessment can be performed within protocol

IRB-approved recruitment flyers are placed in military medical clinics, barracks, and other locations frequented by active-duty military personnel such as coffee shops, restaurants, and fitness centers on and near the base. Participants referred to the site PM are screened via phone or in person depending on how initial contact is made. After the participant expresses interest, the site PM provides a brief overview of the trial and asks participants if they are willing to answer a brief screening questionnaire. Potential participants are informed that their answers will be documented in a secure web system and are asked for their verbal consent prior to any information being collected. Answers to screening questions are not identifiable. However, data from these questions are assigned a screening number. After verbal consent is obtained, screening questions are asked. Persons eligible and interested in the trial after this screen are scheduled for baseline visit 1 (BL1). Those who are ineligible are not recorded other than as a number coded as a “screen failure” in the secure trial database. The only data reported during screening are the reasons for screen failure. No personal identifiers are collected.

### Baseline visit 1

During BL1, the site PM conducts the informed consent process in a private room, explaining the trial in detail using the flowchart and images of the strength, balance, and endurance tests. The informed consent document is reviewed in detail. Questions about trial involvement are answered at this step. Key information emphasized during the informed consent process include the eligibility determination process; the strength, balance, and endurance tests; the randomization process; potential risks and benefits; the voluntary nature of participation; a request for compliance with procedures including attending scheduled appointments; avoiding CC outside trial participation and a review of the disclosure required for the Health Insurance Portability and Accountability Act. Those interested, sign the informed consent document with the PM serving as witness to the signature (Additional file 2).

After receiving the signed informed consent, the PM conducts an interview, collects demographic information, and screens for non-clinical eligibility criteria such as age, active-duty status, and patellar height. All data are recorded on a paper form. At the end of this visit, participants deemed eligible based on the interview are scheduled for an eligibility exam by the trial chiropractor. This may occur on the same day as the informed consent process and baseline interview. However, in most circumstances, it occurs on a separate day following this visit. Data from the interview are entered directly into STaRS from the paper form.

### Eligibility exam

Following the baseline interview, eligible participants receive a clinical evaluation from the trial chiropractor. This examination determines a working diagnosis, screens for exclusion criteria (such as potential safety risks in performing the strength, balance, and endurance tests or the need for a referral to another provider). When available, other health records are reviewed. As part of the safety assessment, participants are asked to assume the positions needed for the balance and strength tests. Individuals unable to perform this task due to pain or safety reasons because of their clinical status are not eligible. Likewise, those unable to perform the endurance test due to pain or another clinical reason are ineligible. The eligibility exam may be performed on a separate day due to schedule availability. However, it is still considered part of BL1. Clinically based exclusion criteria related to test performance safety, though otherwise not a contraindication for CC, include presumptive or confirmed nerve root compression and imaging evidence of neuroforaminal or central canal stenosis. Exclusion criteria representing conditions best managed by another health discipline rather than CC include LBP from a visceral pain source, spinal pathology, chronic pain syndrome, and inflammatory spinal arthropathy.

### Eligibility confirmation

Following the eligibility exam, the trial chiropractor and PM meet to verify the eligibility criteria for each participant. This step is part of a formally structured eligibility determination process developed by our team, which includes trial personnel in separate and distinct roles [29]. The eligibility confirmation step is a defined time point for the PM and chiropractor to formalize, justify, and confirm eligibility decisions based on standardized operational definitions. This step serves as a quality control measure intended to prevent drift of eligibility definitions during the trial. The process includes procedures for consulting with investigators if or when disagreement occurs or when the specific application of the eligibility criteria is unclear. The eligibility criteria are systematically reviewed by the chiropractor and PM, with decisions documented on a paper form. The PM then enters the eligibility information into STaRS. After confirming eligibility, the PM contacts each participant individually to inform them of their status and to schedule baseline visit 2 (BL2) for those who are eligible.

### Baseline visit 2

BL2 occurs within 30 days of BL1. If allocation does not occur within 30 days, the eligibility exam and consent process are repeated for those who still wish to participate. BL2 begins with a PM-administered safety check, in which the PM asks if, since BL1, the participant has experienced any injury, change in health status, or worsening LBP. The PM also asks questions pertaining to medication use and treatments received for LBP within the past week. If there are no negative changes in participant status, the PM asks the participant about PROs and initiates the strength, balance, and endurance testing. Any worsening in participant health status necessitates a follow-up assessment with the chiropractor to re-evaluate test performance safety. In these instances, the trial chiropractor performs a clinical interview and examination, if necessary. When clinical re-evaluation is needed, testing does not commence unless documentation from the chiropractor indicates the participant may safely proceed.

PROs are collected at BL2 prior to allocation and baseline strength, balance, and endurance testing. Participants complete PROs through STaRS. The PM provides each participant with a unique username and password, which are generated by STARS prior to BL2 and are used to access questionnaires. Upon accessing STARS, each participant is required to reset their password. Participants may then complete the questionnaires. PROs include a numerical rating scale for pain intensity (0–10), the Roland Morris Disability Questionnaire, bothersomeness of symptoms questionnaire, PROMIS-29, Fear-Avoidance Beliefs Questionnaire, a global improvement question, and questions about expectations for care and physical activity outside of work duties. Approximately 10 min is needed to complete the PRO questions.

Following PROs, all participants undergo baseline strength, balance, and endurance testing. At each test session, participants first perform the balance test, followed by the strength test, and conclude with the endurance test. This test order prevents the endurance test, which can cause fatigue in trunk and lower extremity muscles, from influencing the short-term maximum strength and balance outcomes. A detailed description of each test is given in the section on outcome measures. Participants are guided through each test using a detailed checklist to ensure standardized performance and data collection.

### Allocation

At the completion of BL2, participants are allocated and scheduled according to group assignment. Group allocation occurs through a 1:1 ratio by a predetermined, computer-generated, restricted randomization scheme with random block sizes of 2, 4, and 6. A trial biostatistician generated the allocation sequence. The PM accesses the treatment allocation module within STaRS by selecting the participant’s ID and initiating the allocation process, which produces a coded group assignment. Group assignment, personnel ID, date, and allocation time point are stored in the database. Participants are allocated to either CC or a waiting list. Once allocated, participants are verbally informed by the PM. Future group assignments are concealed from trial personnel. If the web system cannot be accessed, a back-up system of predetermined, sequentially numbered opaque envelopes is available. Data analysts and the PI are blinded to group assignment.

### Trial interventions

Participants in the CC group receive evaluation and treatment from a chiropractor over a 4-week period. The number of chiropractic visits per participant is tracked throughout the trial. CC participants are asked to refrain from receiving CC or SM by any other provider during the 4-week intervention period. CC and waiting list group participants may receive other care available through the military health system. In weekly phone calls with the PM, participants are asked to self-report any health care received. Self-reported care is confirmed via a review of their military health record.

CC participants receive SM. SM procedures are broadly divided into two types: thrust and non-thrust [30]. Thrust SM is a high-velocity low-amplitude procedure characterized by a single short duration thrust (ranging from 100 to 500 ms) of low amplitude force applied to a target joint that often results in an audible sound or cavitation [31–33]. Non-thrust SM employs low-velocity and often repeated joint movements of varying amplitude [34, 35]. Though the application of thrust and non-thrust SM procedures vary from each other in terms of biomechanical characteristics, both types target primarily joints, whereas many other manual therapy techniques target muscles, fascia, or other soft tissues [36]. If no SM treatment is clinically indicated, it is not provided. Care delivered is tracked throughout the trial by the PM by accessing the electronic medical record system. After the completion of each appointment, the PM obtains a paper form from the trial chiropractor and transcribes the treatment information into STaRS.

The original protocol called for all CC participants to receive a brief functional movement screen with the intention of providing specific exercises based on screening results [37–39]. However, due to a change in the treating clinician partway through the trial, seven participants received this screen but only two were confirmed to have received exercise recommendations based on this screening. The protocol was subsequently revised to remove the brief functional movement screen. Cryotherapy (ice or cold packs) or heat are recommended based on individual clinical findings.

### Waiting list

Participants allocated to the waiting list group are asked to avoid chiropractic treatment or SM by any other provider within or outside the military health system during the active trial period. Participants in this group are scheduled for one additional visit 4 weeks (±7 days) after allocation. Waiting list group participants may receive any other care available to them. All treatment obtained for LBP during this trial is documented via participant self-report and confirmed through review of their military medical record. After trial completion, waiting list group participants are offered the option to be scheduled for CC. Care provided beyond this time is not part of the trial and no data are collected. During weeks 1–3, the PM contacts each participant (both groups) weekly to track treatments for LBP and to screen for adverse events.

### Final visit

The final visit is conducted 4 weeks (±7 days) from allocation. It begins with another safety check prior to testing. Participants then complete PROs by logging into STaRS on the dedicated trial computer. Balance, strength, and endurance tests are performed next. At the end of the visit, participants are thanked for their participation and waiting list group participants are offered the option to receive CC. For those who desire care, the PM facilitates contact with the chiropractic clinic for scheduling.

### Outcome measures

**Fig. 2:**
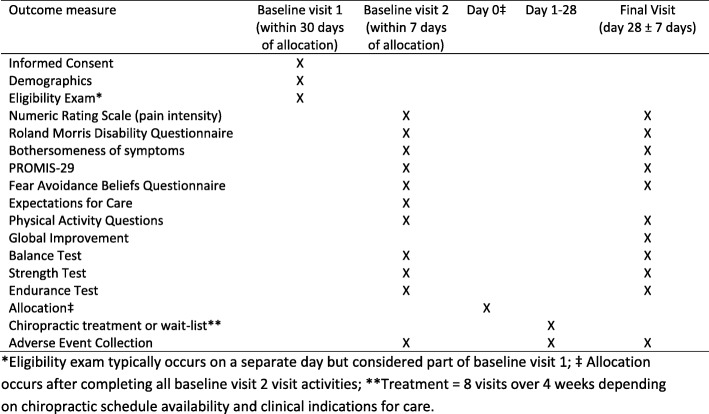
Schedule of events

The primary outcome for this trial is a change in strength following the 4-week active period. Strength was chosen as the primary outcome because it is the muscular function most fundamentally necessary for balance and endurance. Preliminary data were also available to the study team after conducting an unpublished pilot study with the strength test used in this trial. Secondary outcomes include changes in balance and endurance and PROs. Figure 2 displays the schedule of events including the data collection schedule for the primary and secondary outcomes.

### Strength

**Fig. 3:**
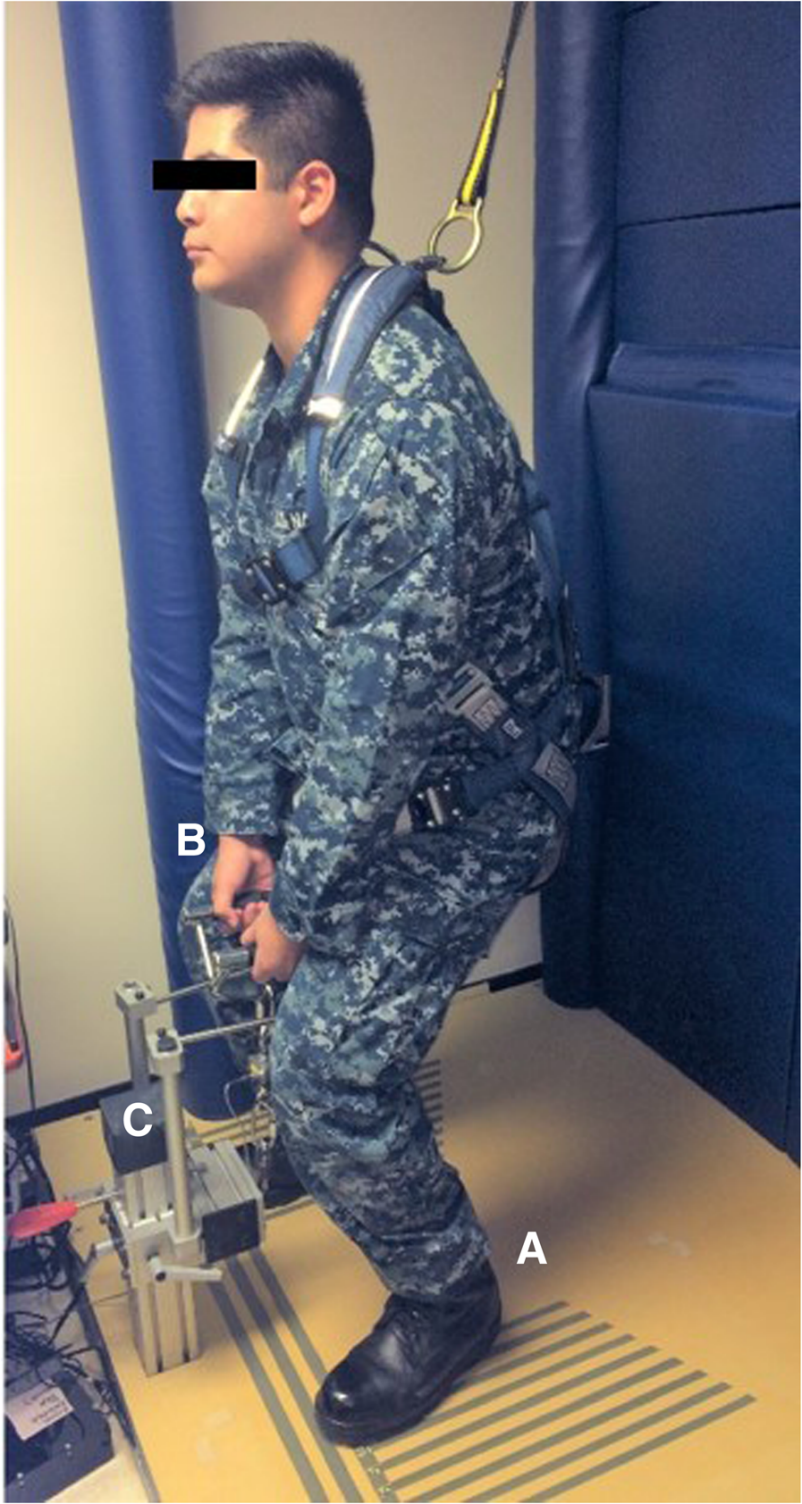
Strength test. Isometric pulling in a semi-squat position with upright torso and eyes facing forward. Foot position is standardized within participants using platform markings (**a**). The bimanual handle (**b**) is attached to a force transducer. Pulling height is standardized using an adjustable post accommodating variable participant height (**c**)

The strength test involves isometric pulling on a bimanual handle while in a semi-squat position, similar to a test described by da Silva and colleagues [40]. The handle is attached to a chain connected to a height-adjustable support anchored to the floor. The chain connecting the handle to the anchored support contains a force transducer (FSH00080, FUTEK Advanced Sensor Tech, Inc., Irvine, CA). The height of the handle is determined by the participant’s patellar height and set by the PM to ensure standardized positioning within and across participants (Fig. 3).

Participants wear a harness allowing free movement of the arms, legs, and torso to prevent an accidental fall. While performing the test, participants are instructed in a stepwise manner to assume a proper lifting position with feet comfortably spread, knees bent, upright spine, arms perpendicular to the floor while holding the lift handle, and eyes looking straight ahead.

Participants are instructed to pull first with a self-monitored light force to confirm they are in the right pulling position and to assess for increased pain. Pulling occurs for less than 5 s with a gradual release of pressure to end the test. Participants are instructed to stop pulling if any increased discomfort is experienced. If the light pull is successfully accomplished without increased discomfort, participants pull again with a self-monitored medium force. If this occurs without increased discomfort, a maximum-effort pull test is attempted. The maximum pulling force is recorded in pounds (lbs.); 4.45 newtons equals 1 lb. If the final pull is halted before reaching the maximum effort, the highest (peak) pulling force is used for the data analysis.

During the maximum pull test, participants are instructed to increase the pulling force gradually up to their maximum without increasing discomfort. The maximum pull procedure is repeated three times with a minimum 2-min rest period and a voluntary warm-up guided by the PM before each test. The warm-up consists of walking within the room, gently bending and twisting the trunk, practicing the semi-squat lifting position, and performing light knee raises and heel kicking motions. The warm-up lasts approximately 3 min. The maximum pulling force recorded is used for data analysis. The stance width for this test is determined by allowing the participant to select the most comfortable position. The handle height is set at 2 in. above the superior aspect of the patella (kneecap). For consistency, the stance width and lifting handle height are recorded during the first assessment and applied at the following assessment. Data are collected and saved to a text file (ASCII format) and converted to Microsoft Excel. The PM saves the test file with the proper identifiers and then uploads it to the secure web system.

### Balance

To minimize potential carryover effects from the strength or endurance tests, the balance test is performed first. The one-leg standing test is an assessment of postural stability [41]. To prevent the remote possibility of falling, participants wear a safety harness that allows free movement of the arms, legs, and torso. Participants perform this test without shoes.

**Fig. 4:**
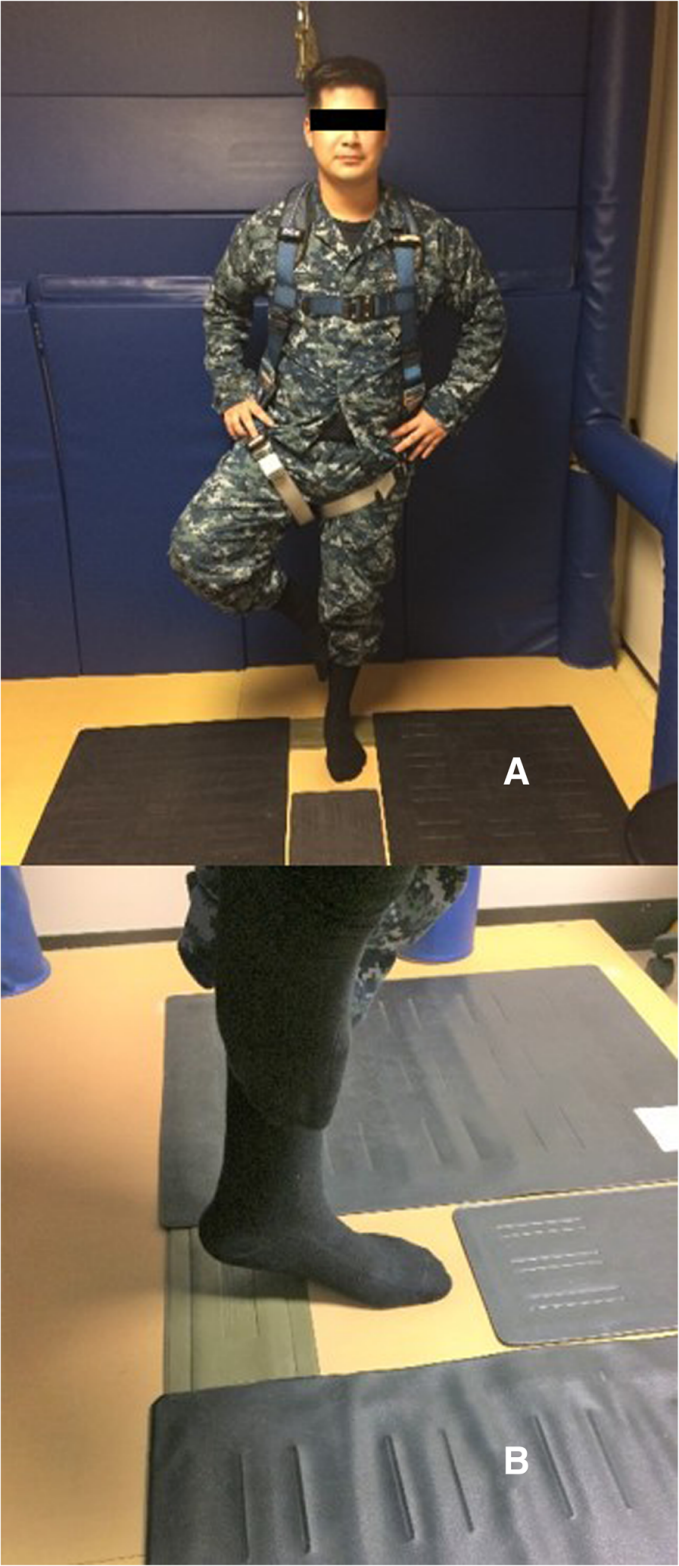
Balance test. Test performed with eyes open and with eyes closed. Pressure sensitive pads (**a**) are connected to a computer timer. Lifting the heel (**b**) removes the pressure, which starts the timer. Touching any pad with either foot stops the timer and ends the test

First, participants are allowed to practice the balancing position by placing both hands on the iliac crest region. Their dominant kicking foot is placed against the supporting leg below the knee (Fig. 4). Raising the heel of the supporting foot completes the balance position. The test commences when the heel is raised from the floor. A sensor pad under the heel activates a computer timer. The timer stops when either the heel of the supporting foot or any part of the other foot touches the floor. The balance test is repeated three times with eyes open and then three times with eyes closed. The longest holding time, recorded in milliseconds, under each condition is used for data analysis [41]. Data including appropriate identifiers (e.g., ID, test date, etc.) are saved in a text file (ASCII format), copied into Microsoft Excel, and uploaded to STaRS.

### Endurance

**Fig. 5:**
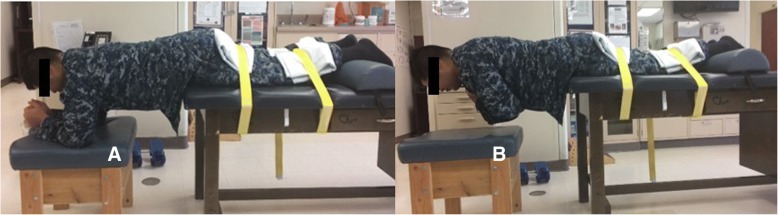
Endurance (Biering-Sorensen) test. **a** Head, shoulders, and trunk extend over the edge of a table. The lower extremities are strapped to the table. **b** Timing begins when the participant lifts their arms off the support stool while maintaining a neutral trunk position. The test ends if pain or discomfort is experienced or after a maximum of 180 s.

The Biering-Sorensen test is an assessment of trunk muscle endurance used as an outcome measure in clinical and research settings because of its low cost, safety, reliability, and ease of use. The test is performed efficiently with minimal specialized equipment [42, 43]. As displayed in Fig. 5, participants lie face down on a padded table. The head, shoulders, and trunk extend over the edge of the table. Initially, they support themselves by resting their forearms and elbows on a padded cushion. The lower extremities are strapped to the table with wide straps as firmly as comfort will allow at the upper thigh and slightly above the ankles. Participants lift their arms off the support and maintain a neutral trunk position for as long as possible. The test ends if pain or discomfort is experienced, if the neutral trunk posture is not maintained, or when the test reaches 180 s. The holding time is recorded in seconds with a handheld stopwatch. The reason for ending the test is also recorded (e.g., discomfort, fatigue, time limit, etc.). Because this test induces trunk, pelvic, and thigh muscle fatigue, it is performed after the balance and strength tests. The test time is recorded on the testing checklist and submitted to STaRS.

### Patient-reported outcomes

All PROs are collected at BL2 prior to baseline testing (except global improvement) and group allocation. All PROs, except expectation of care, are collected again at the final visit. Pain severity is collected using an ordinal 11-box numerical rating scale (0 = no pain to 10 = worst imaginable pain) for the worst and average levels of LBP over the past 24 h [44]. LBP-related disability is collected using the Roland Morris Disability Questionnaire, a reliable and validated single-page questionnaire commonly used in clinical research [45, 46]. The bothersomeness of symptoms of LBP during the past week is measured using an existing six-question instrument with a 1–5 scale (1 = not at all bothersome to 5 = extremely bothersome). Bothersomeness questions have good internal consistency, construct validity, and responsiveness to change over time in patients with LBP and sciatica [47, 48].

PROMIS-29 is a set of short forms containing measures of depression, anxiety, physical function, pain interference, fatigue, sleep disturbance, and satisfaction in social roles [49]. Each of the seven topic domains contains four questions. With an additional pain intensity rating, there are 29 questions in total. Perceived LBP improvement is assessed with a single question adapted from a study of expectations of acupuncture and massage among patients with LBP [50]. Perceived global improvement is rated on a seven-point Likert scale with anchors “completely gone” to “much worse” [51]. Five questions asking about physical activity outside job duties are also asked.

Most PROs measure different aspects of clinical improvement. Two additional measures collected in this trial identify factors that could potentially influence performance on the strength, balance, or endurance tests. The Fear-Avoidance Beliefs Questionnaire is a 16-item validated instrument measuring back-pain fears on two subscales related to physical activity and work. Ratings span from 0 (completely disagree) to 6 (completely agree) [52–54]. Because expectation can contribute a significant non-specific effect [50], all participants answer two questions about their expectation of the benefit of CC and the overall improvement over the next 4 weeks prior to allocation.

### Data collection and management

#### Data collection

Data are collected at each stage of recruitment, allocation, and throughout treatment, to ensure reporting according to the Consolidated Standards of Reporting Trials guidelines [55]. We collect recruitment source, total number of responses per recruitment source, the disposition of each potential participant (i.e., screening failure, ineligible, does not wish to participate, or allocated), the number allocated, participant compliance to treatment protocol, the number lost to follow-up, and the number of participants completing the trial.

Electronic data capture through STaRS occurs via unique login credentials for each user. Login credentials designate and direct access to the appropriate application areas within STaRS. PMs enter BL1 and BL2 data into customized logic-based electronic forms with embedded validation checks to ensure eligibility status prior to allocation. PRO data collected at BL2 and the final visit are entered into STaRS. Balance and strength test data are recorded electronically and uploaded to STaRS. Endurance test data are recorded on paper forms and transcribed to STaRS.

The Office of Data Management at the PCCR serves as the data coordinating center. STaRS is an integrated participant database and permissions system controlling individual access to web modules. STaRS is programmed to support data and project management and includes full technical support. It has procedures for data security, data quality control, storage, and back-up. STaRS includes modules for baseline screening; eligibility checks; treatment allocation; participant tracking; generating reports; and data file transfers for the balance, strength, and endurance tests. Uploaded files are downloaded by the data core manager and stored on a secure server at the PCCR.

All data are stored on a secure internal server running Microsoft SQL Server. STaRS resides on a production web server (IIS V10.0) secured with Secure Sockets Layer (SSL) 256-bit encryption. The system is maintained by the Palmer College of Chiropractic Information Technology department. Select personnel from the Office of Data Management have access to the SQL tables via a Microsoft SQL Server Enterprise Manager interface. STaRS is programmed in ASP.NET v4.0 in C# and SQL using Microsoft Visual Studio 2010. Servers reside behind a stateful firewall. To perform the data cleaning procedures, the data core manager will perform a soft database lock (Microsoft SQL server) and use programs written in SAS System for Windows (Release 9.4) using SAS ACCESS. After all data have been collected, and edits are recorded and performed, a final database lock will be implemented, removing all update access to the database. Final datasets for analysis and the data dictionary will be created by the data core manager, who will write and test SAS programs from the final locked database.

Paper forms containing participants’ names (informed consent documents) and data collection forms containing unique trial ID numbers are stored in locked filing cabinets accessible by the PM. At trial completion, informed consent documents will be stored at NHP for 6 years. Paper data collection forms will be transferred to the PCCR and stored in a secure room for up to 7 years.

### Statistical methods

We will use an intention-to-treat approach in which participants with non-missing data will be analyzed according to their assigned treatment. We will use SAS version 9.4 (SAS Institute Inc., Cary, NC) for data analysis. We will calculate descriptive statistics for baseline characteristics and for maximum strength, balance, endurance, and PROs at baseline and week 4 for both groups. Changes in outcome variables from baseline to week 4 will be analyzed with an analysis of covariance (ANCOVA), adjusting for the baseline value of the respective outcome variable centered at its mean and other covariates that are not balanced between groups. The group mean changes and between-groups differences in mean change based on the ANCOVA will be reported with 95% confidence intervals.

### Sample size

We conducted a power analysis using Proc Power in SAS for our primary outcome variable, maximum strength. We used the standard deviation of 48 lbs. observed in our pilot study and assumed that maximum strength will remain unchanged in the waiting list control group. A total sample size of 82 participants, with 41 per group, provides 90% power to detect at least a 20% difference in mean change between groups at a 0.05 level of significance. In another clinical trial conducted by the investigative team at this site, we observed 23% missing data at week 2 and 29% at week 4 [56]. Therefore, we increased the sample size to 110, with 55 per group, to account for the possibility of a 25% dropout rate.

### Internal quality assurance

Data management and quality control of data entered into web forms are performed using SQL views, stored procedures, and real-time web-based reports. Automated reports are viewed by the data core manager and PMs and guide the need for improved documentation, protocol revisions, or personnel retraining, and to track recruitment and participant accrual.

The lead PM audits the trial site quarterly. Audit procedures are designed to maintain data integrity and ensure protocol fidelity. The lead PM reviews regulatory and informed consent documents. Paper documents are verified against electronic data entered into STaRS. Errors discovered are documented, corrected, and reported to the PI, collaborating investigators, and appropriate regulatory bodies when applicable. Site visits also include meetings with the site PI and chiropractor to facilitate communication, discuss timelines, and address concerns. Key information from site visits is communicated to co-investigators by the lead PM.

### Study event monitoring and reporting

#### Study events

Study events are classified into three categories: (1) protocol deviations, (2) protocol violations, and (3) unanticipated events. A protocol deviation is any variance in the approved trial protocol, criteria, or procedures that does not affect a participant’s safety, rights, or welfare or the integrity of the trial and its resultant data. Protocol violations are deviations that increase risk, decrease benefit, or affect a participant’s rights, safety, or welfare or the integrity of the resultant data. Events that do not meet the above definitions but may affect a participant’s rights, safety, or welfare or the integrity of the resultant data are classified as unanticipated events.

All study events are entered into STaRS by trial personnel. The lead PM and PI receive an automatic email notification when a study event is submitted. These individuals are responsible for classifying the event and reviewing its details to ensure proper reporting. Submission of information on study events to an IRB is dictated by the reporting requirements for each IRB overseeing the trial. Deviations that may affect the safety or rights of participants, or the integrity of the trial, are promptly reported to the DSMC and the U.S. Army Medical Research and Material Command Human Research Protection Office.

#### Adverse events

An adverse event is any untoward medical occurrence that happens during the conduct of the trial and which may have a causal relationship with a trial procedure [57]. A serious adverse event is any adverse experience occurring during the trial period that results in any of the following outcomes: death, a life-threatening adverse experience, hospitalization or prolongation of existing hospitalization, a persistent or significant disability or incapacity, or a congenital anomaly or birth defect. We monitor adverse events from when consent is signed until the final visit.

The PM inquires about adverse events weekly via telephone. Participants are also instructed to contact the PM if they experience significant pain, discomfort, or distress that they believe may be associated with treatment. Adverse event reports are electronically documented and submitted by the PM through STaRS. The lead PM and central trial clinician receive an automatic notification email for each adverse event report. The central trial clinician classifies adverse events, which are then reviewed by the lead PM. Classification occurs on three levels: (1) seriousness, (2) expectedness, and (3) relatedness. Following adjudication, adverse events meeting IRB reporting criteria are reported to each relevant IRB. Additionally, all adverse events that may affect the safety or rights of volunteers are reported to the DSMC and the U.S. Army Medical Research and Material Command Human Research Protection Office as required. Events not meeting the criteria for reporting are submitted to the IRBs at the next review or otherwise as required.

### Trial limitations

Studies using a waiting list as a control are limited because participants are not blind to group and waiting list participants do not have the same attention given to them as those in an active intervention arm. We chose a waiting list as the control because inert sham treatments for SM procedures involving the low back have not been sufficiently developed to be used widely in clinical trials without participants recognizing the procedure as sham and because clinical outcomes are secondary to objectively measured strength, balance, and endurance. Further, sham treatment was not an available treatment option at this treatment facility, making a waiting list option the only practical and available control group strategy.

CC participants may not receive the same treatment or the same number of visits as each other. This limitation is due to the varied nature of conditions comprising LBP and to scheduling limitations at the health facility. The variation in both treatments and visits likely to be experienced by participants reflects CC as applied in U.S. military health treatment facilities. Thus, the results are more likely to represent the effects of CC as applied in these settings. CC was intended to be provided by a single practitioner. However, this plan was altered shortly after recruitment began. Only two chiropractors provide treatment in this trial, with most care administered by one. The lack of practitioner and participant blinding to group is a limitation common to studies involving manual therapies. The PM functions as the assessor for the strength, balance, and endurance tests. To minimize this limitation, tests are conducted using scripts and checklists to ensure the actions and language are consistent among participants to minimize the possibility of communicating disparately between group members.

### Challenges

There have been several challenges in conducting this trial to date, leading to periods where study activities, including recruitment, were temporarily suspended. One month after recruitment launched in April 2016, the site PI received deployment orders, which led to the suspension of study activity. After a replacement PI was identified and regulatory approval obtained, recruitment resumed in June 2016. In October 2016, enrollment was again suspended when the study chiropractor unexpectedly became seriously ill and passed away. A period of 9 months elapsed before a new chiropractor was able to engage in patient care and recruitment restarted. This lapse consisted of the subcontractor candidate search, interview and hiring process, employee credentialing, and NHP facility-specific training on institutional policies, procedures, and electronic health record use. In addition, the second PI was transferred to another site, resulting in a second temporary suspension lasting until June 2017. This may impact our ability to reach our targeted sample size of 110. However, if this is the case, we do not anticipate an underpowered study because as of 8 October 2018, only 6.1% of participants have withdrawn or dropped out, rather than the anticipated 25% that was used to inflate the necessary sample size to 110.

## Discussion

In this trial, we will evaluate the effects of CC on measures of strength, balance, and endurance, potentially important factors leading to reduced readiness in affected military personnel. These objective measures were chosen because of their potential to help understand how these key aspects of fitness relate to military personnel with LBP and are influenced by CC. Upon completion of this trial, the authors plan to publish the study results in a peer-reviewed journal. Results from this trial may inform further investigations of neuromuscular mechanisms and the potential use of objective measures for assessing clinical improvement for conditions causing LBP.

### Trial status

The first participant was allocated on 26 April 2016. As of 8 October 2018, 93 participants had been allocated. The last allocation is expected to occur in December 2018 followed by a 4-week active trial period.

## Additional files

Additional file 1: Consent form and participant information sheet. (PDF 73 kb)

Additional file 2: SPIRIT 2013 Checklist: Recommended items to address in a clinical trial protocol and related documents. (DOCX 20 kb)

## Abbreviations

BL1: Baseline visit 1
BL2: Baseline visit 2
CC: Chiropractic care
DSMC: Data safety monitoring committee
IRB: Institutional review board
LBP: Low back pain
NHP: Naval Hospital Pensacola, FL
PCCR: Palmer Center for Chiropractic Research
PI: Principal investigator
PM: Project manager
PROMIS: Patient-Reported Outcomes Measurement Information System
PRO: Patient-reported outcome
RCT: Randomized controlled trial
SM: Spinal manipulation
STaRS: Submission Tracking and Reporting System
