# Supplementary material for: Assessment of chiropractic care on strength, balance, and endurance in active-duty U.S. military personnel with low back pain: a protocol for a randomized controlled trial
Source: Trials. 2018 Dec 5;19:671. doi: 10.1186/s13063-018-3041-5 (PMC6280458; doi:10.1186/s13063-018-3041-5)
Supplement: Supplementary file 1 — Consent form and participant information sheet. (PDF 73 kb) [file 13063_2018_3041_MOESM1_ESM.pdf]

Study Expiration Date	13 Mar 19
CID Version	8
Protocol Version	4
Protocol Date	February 2018

NAVAL HOSPITAL PENSACOLA
Pensacola, Florida

CONSENT BY A SUBJECT FOR VOLUNTARY PARTICIPATION IN A CLINICAL INVESTIGATION

SUBJECT ID # __________ **DATE: __________**

Please read this form carefully. Take time to ask the study doctor or study staff as many questions about the study as you would like. If there are any words or information that you do not understand, the study doctor or study staff will explain them to you. Reading this form and talking to the study doctor or study staff may help you decide whether to take part or not. Before you take part in the research study, you must sign the end of this form.

1. Study Title:

You have been asked to voluntarily participate in a research study entitled **NHPC.2015.0003** "**Assessment of Chiropractic Treatment: Strength and Balance (ACT 3)**," being conducted at the Naval Hospital Pensacola, Pensacola, Florida by medical researchers from the Naval Hospital Pensacola, NBHC-NATTC, RAND Corp., the Palmer Center for Chiropractic Research, and the Samueli Institute. The DoD is providing funding for this research.

2. Why Is This Study Being Done?

The purpose of this study is to measure the effects of chiropractic care on strength and balance in active duty soldiers with low back pain. Strength and balance are important measures of military readiness.

This study will compare strength, balance, and endurance (a measure of muscle strength) in participants that receive 4 weeks of chiropractic care with participants who do not receive chiropractic care.

3. Why Are You Being Asked To Take Part?

You are being asked to take part in this study because you have low back pain and are an active duty service member of the U.S. Armed Forces.

This study includes only those people who choose to take part. Please take your time to make your decision and feel free to ask any questions that you have.

4. Screening Process to Qualify for Participation in This Study

Some tests must be done and some information must be collected before the Investigator can confirm that you meet the qualifications to become a subject in this study. This is called the "Screening Process". These tests may have been done or this information collected as a part of your regular medical care.

5. What Is Involved In This Study?

This study is called a randomized controlled trial. If you choose to take part in the ACT 3 study, you will be randomly assigned to one of 2 groups: Group 1 or Group 2. This means you will be assigned into one of these groups by chance (like flipping a coin). In this study, a computer program is used to determine which group you are in. Neither you, your study doctor, nor the research staff will be able to choose the group to which you are assigned.

- Group 1 *will receive* chiropractic care for 4 weeks after group assignment. These 4 weeks are called the "active study period."
- Group 2 will *not* receive chiropractic care during the active study period but will be able to receive chiropractic care after those first 4 weeks.

If you take part in this study, you will take part in the following tests and procedures:

Baseline visit 1

The baseline visit will last about 1.5 hours, which may be split into 2 separate days, and will consist of the 1) informed consent process 2) interview with a researcher, and 3) low back exam by a study doctor.

- **Informed consent process**: A member of our study team will provide you with a detailed review of the study and the procedures and tests that are part of the study. You will be given time to read the consent form and discuss any questions or concerns you have about taking part in the study.
- **Interview**: After the informed consent process is completed, a member of the study team will ask you basic questions about yourself, your low back pain, treatment you are receiving for your low back pain, and other questions to see if you are eligible for the study.
- **Low Back Pain Exam**: A study doctor of chiropractic will review your health history and conduct an exam to see if you can safely receive chiropractic care and safely perform the research tests. This exam will include asking you about your back and neck, other joints and muscles, previous treatments, and your health status. The doctor also will perform a standard exam on your back and other joints and muscles to help establish a diagnosis.

After your baseline visit 1, the study doctor and study project manager will review your interview and low back pain exam results to decide if you are eligible to participate in the study. The study project manager will then call you to inform you of your status. If you are eligible, you will be scheduled for a baseline visit 2. If you are not eligible, you still will receive a phone call from the study manager to inform you of your status. *You may still be eligible to receive chiropractic care even if you do not qualify for the study.*

Baseline visit 2

The baseline visit 2 will last about 1-1.5 hours. You will be asked to answer questionnaires and perform research tests that measure your strength, balance, and endurance (another test of muscle strength).

- **Questionnaires:** You will be asked to answer questions about your past and current health, low back pain, physical activity habits, and basic background information. You also will complete standard research forms about your low back pain and how it affects your daily activities and quality of life. These questions will be answered on a computer.
- **Research tests:** You will be asked to complete the strength, balance, and endurance tests (described below). These tests will be completed twice during the study; once at the baseline visit 2 (before group assignment) and again at the end of the study.

Group Assignment
After you complete the baseline visit 2, you will receive your random group assignment. The computer will tell us what group you are in. The assignment is random; like a flip of a coin. Your chances of being assigned to each group are equal. That means half the participants in this study will be in Group 1, and half the participants will be in Group 2. Neither you nor any member of the research staff can choose your group.

If you are placed into Group 1, you will receive chiropractic care for 4 weeks after group assignment. If you are placed into Group 2, you will wait 4 weeks to see the chiropractor.

Description of Study Groups

There are no experimental or unapproved treatments in this study. Chiropractic care is an approved treatment for low back pain. You may also start or continue with any other treatment for your low back pain during the study.

Group 1: If you are assigned to Group 1, you will be scheduled with the study chiropractor for 2 treatments per week for 4 weeks. This schedule may vary. Each visit lasts about 15-30 minutes. For this study, chiropractic treatment may consist of manipulation to your spine. Manipulation is the process of moving the body (back, arms, legs) through different positions to correct improper function in those areas. Some people call these manipulations "adjustments". Manipulation can occur quickly (you may hear a popping sound) and sometimes it may be slow (usually no popping sound). The study doctor will explain all procedures prior to performing them. The study doctor will also determine if other treatments may be helpful to you like heat or ice therapy. Your study doctor will also give you exercises to help you treat your low back pain. It is possible that on some visits you will not receive any treatment.

Group 2: If you are assigned to Group 2, you will wait four weeks before you may receive chiropractic care. After you complete the final set of research tests at 4 weeks, you will be able to receive chiropractic care if you wish. Your study manager will help schedule you for chiropractic care at that time.

Final study visit
The final visit will occur about 4 weeks after group assignment. During this visit, you will complete research questionnaires and perform the same strength, balance, and endurance tests that you completed at the baseline visit 2. These tests are described in detail below. This visit will last about 1-1.25 hours.

- **Research questionnaires**: Before you complete your strength, balance, and endurance tests, you will be asked to answer questions like the questions you completed at your baseline visit. These questions will ask you about your low back pain and how it affects your daily activities and quality of life. These questions will be answered on a computer. These questions will take about 20 minutes to answer**.**

Description of Research Tests
A member of the study team will ask you if your health status has changed before you begin the study tests. If your health status has changed, you may be asked to see the study doctor before performing the study tests to be sure it is still safe for you.

Balance test: You will perform the balance test first. The goal of this test is to see how long (length of time) you are able to stand on the ball of foot with your eyes open and then with your eyes closed. For the balance test, you will wear a safety harness and place your hands on your hips. We will ask you to place one foot against the inside of the other (supporting) leg. We will then ask you to raise the heel of the supporting leg off the floor and balance for as long as you can. The test starts when you raise the heel (that is on the floor) off the platform to balance on the ball of your foot. The test will stop when you are unable to hold this position or the heel of the supporting foot touches the floor. You will be given a chance to practice before you start the test. This test will be performed 3 times with your eyes open and 3 times with your eyes closed. You can rest in between each test.

Strength test: The goal of this test is to measure how hard you can safely pull on a handle that measures how hard you can pull. You will stretch and warm-up before you start this test. For the strength test, you will again wear a safety harness. You will be asked to bend your knees and get into a semi-squat (near squat) position. We will show you how to do this and make sure you are in the correct position. Then you will be asked to slowly pull up on a handle attached to a device that measures how hard you are pulling. You will do a series of 3 pulls. The series of pulls is so that you get used to the test and to make sure you do not pull so hard it increases your pain. We will first ask you to pull on the handle with a light force and then slowly release the handle. We will check in with you after each pull to make sure that you are not feeling any increased discomfort. You will only move to the next level of pulling if you are not feeling more discomfort and we think it is safe. Next, we will ask you to slowly pull on the handle with your medium force. As soon as you reach your medium pulling force, you will slowly release the handle. We will then ask you to slowly pull on the handle as hard as you can *without causing or increasing pain* and release slowly. After this pull, you will be asked to rest for two minutes. This test (the hard pull) will be done 2 more times with 2 minutes rest between each test.

Endurance test: The goal of this test is to see how long you can hold your upper body in a straight position. For this test, you will be asked to lie face down on a padded table with the upper half of your body extending off the table. A support will be placed on the floor, below your upper body so you can lean on it with your forearms and elbows before and after the test. Your legs will be tightly secured to the table with wide straps (like seatbelts). Securing your legs will allow you to lift your upper body and feel stable during the test. We will then ask you to lift your arms from the support pad and form a straight position with your body (parallel to the floor). The purpose of the test is to hold that position as long as you can without causing any pain or discomfort. The test ends when you place your arms back on the support pad because you are unable to hold a straight position any longer, if you feel any pain or discomfort, or if you hold the position for 180 seconds (3 minutes).

It is important to let the research staff know if you feel any discomfort or pain during any of the research tests so that we can modify or stop the test.

Weekly phone call

After group assignment, you will receive a phone call each week for 3 weeks. We will call you to ask how your back is feeling. We will also ask you about the medications you are taking for your low back pain and any treatments you are receiving.

6. How Many People Will Take Part In This Study?

We anticipate that up to 250 subjects will be consented and screened for eligibility for the study. We will randomize a total of 110 subjects into one of the 2 groups.

7. How Long Will You Be In This Study?

Your participation in this research project will be for a period of 5-6 weeks. The screening process will take about 1-2 weeks. The study treatment period will last about 4 weeks.

You may stop participating in this study at any time. However, if you decide to stop participating, please talk to a study team member. If you decide to stop participating because of a health reason, please talk to a study team member and your regular healthcare provider.

8. When Should You Not Take Part?

In order to participate in this study, you must be active duty military.

You should not take part in this study if:

- You have knowledge of deployment or transfer of duty station within the study period (5-6 weeks).
- You are seeking a permanent disability status within the military.
- Your study doctor decides you are not a candidate for chiropractic care.

- Your study doctor decides it is not safe for you to perform the study tests or participate in the study.
- You are a woman and you are planning to become pregnant within the next 6 weeks.

9. What Are The Risks Of The Study?

The exams and treatments used in this study involve very little known risk. The possible risks and discomforts from being in this research study may include:

Likely

- Muscle and/or joint soreness – some participants may experience muscle or joint soreness or strain related to the low back exam, chiropractic treatment, prescribed exercises, or from performing the research tests.

Moderate

- Radiating discomfort to arms, hands, legs or feet – Some individuals could feel radiating discomfort following chiropractic treatment or from pulling too hard during the strength test. Some individuals may also feel tingling in the low back, thighs, or legs. These symptoms usually go away on their own in a short period of time.
- Exposure to low doses of radiation - It is possible that the study doctor will order x-rays of your low back. If so, you will be exposed to a small amount of radiation.

Rare

- Falling in a harness during the research tests
- Bone fracture from falling, or excessive strain during the strength test
- Nerve injury that may cause loss of bowel or bladder function, lower body sensation, or leg paralysis

These risks are small. If our study doctor identifies you at a high risk for any of these safety concerns, you will not be eligible for the study or you may be withdrawn from the study because it is no longer safe for you to participate. We screen our participants carefully to ensure that only people who meet the study criteria and safety guidelines are enrolled in this study.

While involved in this study, you may be at risk for the side effects listed above. If you have any questions, you should discuss them with the study doctor and/or the healthcare provider of your choice. Other medications may be given to make the side effects less serious and make you more comfortable. Many side effects go away shortly after treatment or procedures are stopped, but in some cases the side effects can be serious, long lasting or permanent.

Reproductive risks: There are no reported risks to your unborn baby associated with chiropractic treatment if you become pregnant. However, pregnancy can change the low back condition and this study is not designed to look at these changes. The risks for pregnant women performing the strength, balance, and endurance tests are unknown. You should promptly advise the study team listed below if you know that you are now pregnant, if you want to become pregnant during the study period, or if you discover that you have become pregnant during your participation in the study.

There also may be other side effects that are unknown and we cannot predict. For more information about risks and side effects, ask the investigator CDR John Biery at 850-452-8970 ext 139 or contact the study project manager, Crystal Franklin, at 850-377-9183.

10. Are There Benefits To Taking Part In This Study?

If you agree to take part in this study, there may or may not be direct benefit to you. There is no guarantee that you will personally benefit from taking part in this study. We hope the information learned from this study will give us information about the use of chiropractic care and military readiness.

11. Are There Alternatives To This Study?

You may choose to not participate. You and your doctor can discuss the treatment options that are best for you.

12. What About Confidentiality?

We plan to hold all health information in strict confidence, but we cannot guarantee absolute confidentiality. Your personal information may be disclosed if required by law. Authorized personnel from (the Institutional Review Board, Department of the Navy, Department of Defense, Palmer College of Chiropractic, RAND Corp., the Samueli Institute, the FDA, and other agencies, etc.) may have access to your research file for authorized purposes, including verification that your rights have been safeguarded.

Privacy Act Statement

In accordance with the Privacy Act of 1974 (Public Law 93-579), this notice informs you of the purpose of this form and how it will be used. Please read it carefully.

1. Authority. Public Law 104-191; E.O. 9397 (SSAN); DoD 6025.18-R.
2. Purpose. Medical research information will be collected to enhance basic medical knowledge or to develop tests, procedures, and equipment to improve the diagnosis, treatment, or prevention of illness, injury, or functional impairment. This form is to provide the Naval Medical Center Portsmouth/TRICARE Health Plan with a means to request the use and/or disclosure of your protected health information.
3. Use. To any third party or the individual upon authorization for the disclosure from the individual for: personal use; insurance; continued medical care; school; legal; retirement/separation; or other reasons.
4. Disclosure. Voluntary. Failure to sign the authorization form will result in the non-release of the protected health information. This form will not be used for the authorization to disclose alcohol or drug abuse patient information from medical records or for authorization to disclose information from records of an alcohol or drug abuse treatment program. In addition, any use as an authorization to use or disclose psychotherapy notes may not be combined with another authorization except one to use or disclose psychotherapy notes.

   HIPAA: Release Authorization

   a. You have the right to revoke this authorization at any time. Your revocation must be in writing and provided to the facility where the research is being conducted. You are aware that if you later revoke this authorization, the research facility may have used and/or disclosed your protected information on the basis of this authorization.
   b. If you authorize your protected health information to be disclosed to someone who is not required to comply with federal privacy protection regulations, then such information may be re-disclosed and would no longer be protected.
   c. You have a right to inspect and receive a copy of your own protected health information to be used or disclosed, in accordance with the requirements of the federal privacy protection regulations found in the Privacy Act and 45 CFR 164.524.
   d. The Military Health System (which includes the TRICARE Health Plan) may not condition your treatment in MTFs/DTFs, payment by the TRICARE Health Plan, enrollment in the TRICARE Health Plan or eligibility for TRICARE Health Plan benefits on failure to obtain this authorization. (i.e. The MHS may not alter, deny, or make your legal entitlement to benefits a condition of your participation in this study or your decision to provide consent to use your protected health information).

By signing this consent, you are authorizing NMC Portsmouth to obtain and release the information as described in this consent form. You have the right to refuse to sign this permission form.

5. Disclosure. All information contained in this Consent Statement or derived from the medical research study described herein will be retained permanently at Naval Medical Center Portsmouth and salient portions thereof may be entered into your health record. You voluntarily agree to its disclosure to agencies or

individuals identified in the preceding paragraph. You have been informed that failure to agree to such disclosure may negate the purposes for which the research study was conducted.

13. What If You Get Injured?

If you suffer any injury as a result of your participation in this study, medical treatment is available at Naval Hospital Pensacola. All medical care (*including medical treatment for injuries related to this study and medical care unrelated to this study)* will be evaluated and provided in keeping with the benefits to which you are entitled under applicable regulations.

14. Will You Get Paid For Participation?

You will not be compensated for your participation in this study. All treatments related to this study will be provided in accordance with applicable regulations.

15. What Are Your Rights As A Participant?

Your participation in this project is voluntary and your refusal to participate will involve no penalty or loss of benefits to which you are otherwise entitled under applicable regulations. If you choose to participate, you are free to ask questions or withdraw from the project at any time without prejudice to your future care. Any new significant findings developed during the course of the research, which may affect your willingness to participate further, will be explained to you. Please notify the study project manager at 850-377-9183 or the study investigator CDR John Biery at 850-452-8970 ext 139 to ensure an orderly termination process. If you withdraw, you will no longer receive treatments as part of this study. You may still receive treatment as part of your normal course of treatment. Your withdrawal will involve no loss of benefits to which you are otherwise entitled. If you withdraw from this study, your data will be included in the data analysis for this project but only the data collected prior to your decision to withdraw.

16. Can Your Participation In This Study Be Terminated?

The investigator may terminate your participation in this project for the following reasons:

- If being in the study is unsafe or dangerous to your health
- If the investigator feels that it is in your best interest to not participate for any reason
- If you lose your right to receive medical care at a military treatment facility

17. Who Can You Call If You Have Questions Or Concerns About This Study?

If you have any questions regarding this research project, you may contact the study project manager at 850-377-9183 or the study investigator, CDR John Biery at 850-452-8970 ext 139.

A description of this clinical trial will be available on *http://www.ClinicalTrials.gov,* as required by U.S. Law. This Web site will not include information that can identify you. At most, the Web site will include a summary of the results. You can search this Web site at any time.

If you feel you have suffered an injury as a result your participation in this research project or if you have any questions regarding your rights as a research subject at Naval Medical Center, Portsmouth, you can contact the Chair, Institutional Review Board or the Head, Clinical Investigation Department at (757) 953-5939 or DSN 377-5939.

SIGNATURES

Investigators must use the following steps in order to orient the potential subject to the purpose of the research and why they might wish to participate:

- **Step One:** The Investigator must explain the study to the potential subject verbally, providing all pertinent information (purpose, procedures, risks, benefits, alternatives to participation, etc.), and must allow the potential subject ample opportunity to ask questions.
- **Step Two:** Following this verbal explanation, the potential subject should be provided with a written consent form and afforded sufficient time to consider whether or not to participate in the research. "Sufficient time" can range from hours to days, depending on how long it reasonably takes to evaluate the procedures, risks, potential benefits, and alternative treatments.
- **Step Three:** After allowing the potential subject time to read the consent form, the Investigator should meet with the potential subject and answer any additional questions he or she may have.

SUBJECT STATEMENT

By signing below, you are indicating that you were given enough time to read this study consent form, all of your questions about this research project were adequately answered and a copy of this consent form was given to you for future reference. Most importantly, by signing this consent form, you are indicating that you voluntarily agree to participate in this research study.

______________________	______________	______________________
Subject's Signature	Date<br>(DD/MMM/YY)	Typed/Printed Name

INVESTIGATOR STATEMENT

You have explained to the above individual the nature and purpose of the study, the potential benefits and possible risks associated with the study, and the alternatives to participation in this study. You have answered any questions that were raised. You have explained the above to the subject on the date stated on this consent form. Consent was obtained prior to participation in the study.

Investigator participating in / performing consent process

______________________	______________	______________________
Investigator’s Signature	Date<br>(DD/MMM/YY)	Typed/Printed Name
